# Loop Mediated Isothermal Amplification for Detection of *Trypanosoma brucei gambiense* in Urine and Saliva Samples in Nonhuman Primate Model

**DOI:** 10.1155/2015/867846

**Published:** 2015-10-04

**Authors:** Maina Ngotho, John Maina Kagira, Beatrice Muthoni Gachie, Simon Muturi Karanja, Maxwell Wambua Waema, Dawn Nyawira Maranga, Naomi Wangari Maina

**Affiliations:** ^1^Animal Science Department, Institute of Primate Research (IPR), P.O. Box 24481, Karen, Nairobi 00502, Kenya; ^2^Animal Health and Production Department, College of Agriculture and Natural Resources, Jomo Kenyatta University of Agriculture and Technology (JKUAT), P.O. Box 62000, Nairobi 00200, Kenya; ^3^Biochemistry Department, College of Health Sciences, Jomo Kenyatta University of Agriculture and Technology (JKUAT), P.O. Box 62000, Nairobi 00200, Kenya; ^4^Public Health Department, College of Health Sciences, Jomo Kenyatta University of Agriculture and Technology (JKUAT), P.O. Box 62000, Nairobi 00200, > Kenya

## Abstract

Human African trypanosomiasis (HAT) is a vector-borne parasitic zoonotic disease. The disease caused by *Trypanosoma brucei gambiense* is the most prevalent in Africa. Early diagnosis is hampered by lack of sensitive diagnostic techniques. This study explored the potential of loop mediated isothermal amplification (LAMP) and polymerase chain reaction (PCR) in the detection of *T. b. gambiense* infection in a vervet monkey HAT model. Six vervet monkeys were experimentally infected with *T. b. gambiense* IL3253 and monitored for 180 days after infection. Parasitaemia was scored daily. Blood, cerebrospinal fluid (CSF), saliva, and urine samples were collected weekly. PCR and LAMP were performed on serum, CSF, saliva, and urine samples. The detection by LAMP was significantly higher than that of parasitological methods and PCR in all the samples. The performance of LAMP varied between the samples and was better in serum followed by saliva and then urine samples. In the saliva samples, LAMP had 100% detection between 21 and 77 dpi, whereas in urine the detection it was slightly lower, but there was over 80% detection between 28 and 91 dpi. However, LAMP could not detect trypanosomes in either saliva or urine after 140 and 126 dpi, respectively. The findings of this study emphasize the importance of LAMP in diagnosis of HAT using saliva and urine samples.

## 1. Introduction

Human African trypanosomiasis (HAT) is a tropical disease that is endemic in several countries in sub-Saharan Africa. Control of sleeping sickness relies on passive case detection and it is considered to be the most cost-effective when compared to active case detection [1]. Sleeping sickness caused by* T. b. gambiense *is currently responsible for over 90% of all HAT cases [1]. Screening of the population at risk is done by antibody detection with the Card Agglutination Test for Trypanosomiasis (CATT) and confirmed by parasitological methods. Serological tests have varying sensitivities and cannot decisively differentiate between active and cured cases. Furthermore, cured patients can remain CATT seropositive for up to three years due to persisting circulating antibodies, thus prohibiting the use of antibody tests for assessment of treatment success [2]. The parasitological detection techniques also have limitations. The methods are time consuming, tedious, and prone to subjectivity. In addition, low detection rates may occur since* T. b. gambiense* infection is characterized by low parasitemia [3]. False negatives (CATT negative) but parasitemic cases have also been reported [4]. These limitations imply the need for more sensitive and specific diagnosis.

The amplification of DNA has emerged as one of the diagnostic techniques used in studies of infectious diseases [5]. Species specific genes have been used to characterize trypanosomes [6]. The discovery of the* T. b. gambiense*-specific glycoprotein (TgsGP) gene that is specific to the* T. b. gambiense* subspecies heralded its use as a probe for diagnosis. It is the only subspecies-specific gene for* T. b. gambiense* and encodes a 47 kDa VSG-like receptor protein [7]. Amplification of this gene using PCR has successfully been used in clinical samples [8]. However, challenges of the DNA extraction protocols may affect diagnosis of trypanosome infections [9] and requirements of expensive automated thermal cyclers make PCR impractical for adoption in the field [10].

Loop mediated isothermal amplification (LAMP) is performed under isothermal conditions and relies on autocycling strand displacement DNA synthesis [11]. It requires a simple heating device and is rapid and results are easily viewed by several detection formats. The autocycling reactions lead to accumulation of a large amount of the target DNA and by-products such as magnesium pyrophosphate allowing for rapid detection using varied formats. LAMP uses four to six specially designed primers recognizing six to eight regions of the target DNA sequence resulting in a high specificity. It has been used in detection of the* Trypanozoon* subgenus [12],* T. b. rhodesiense* [13], and recently Group 1* T. b. gambiense* [14]. The test has high sensitivity and specificity and does not require specialized equipment, and this makes it a suitable diagnostic test in resource poor settings and would therefore be ideal diagnosis of neglected diseases such as HAT.

The importance of experimental animal models includes controlled conditions and planned sampling among other. The vervet monkey (*Chlorocebus aethiops*) has been developed as a model for early stage HAT caused by* T. b. gambiense *[15]. Using this animal model, the performance of LAMP based on the* TgsGP* gene was assessed in detection of* T. b gambiense* in serum, CSF, saliva, and urine.

## 2. Materials and Methods

### 2.1. Trypanosomes

*Trypanosoma b. gambiense* isolate IL3253 was used in this study. It was isolated from a human HAT patient from Sudan in 1982. The isolate was cryopreserved in liquid nitrogen and for infective purposes the parasites were subinoculated into immunosuppressed donor Swiss mice. At peak parasitemia, heart blood was obtained by cardiac puncture and parasites harvested and diluted to 10^5^/mL using phosphate saline glucose.

### 2.2. Experimental Animals

Six adult vervet monkeys of both sexes weighing between 2.0 and 5.0 kg were used in this study. They were trapped from the wild in an area known to be nonendemic for human trypanosomiasis. The animals underwent a 90-day quarantine during which they were screened for zoonotic diseases and treated for ecto- and endoparasites. They were also trained for ease of adaptation and maintained on commercial pellets (Unga Feeds Ltd., Nairobi, Kenya) supplemented with fresh fruits and vegetables. Drinking water was provided* ad libitum*. The monkeys were housed in stainless steel cages at ambient room temperatures of 18–25°C, under biosafety level II animal holding conditions. At the end of the experiment period, the animals were euthanized by injection with Euthatal (20% sodium pentobarbitone, Rotexmedica^*®*^, Trittau, Germany) via the femoral vein.

### 2.3. Study Design

Six monkeys were infected intravenously with approximately 10^5^ trypanosomes in 1 mL of phosphate saline glucose. The infected monkeys were monitored for a total period of 180 days after experimental infection. Parasitaemia was estimated daily using methods previously described using the rapid matching method [16] and haematocrit centrifuge technique [17].

### 2.4. Sample Collection

The monkeys were anaesthetized on weekly basis with ketamine hydrochloride (Rotexmedica, Trittau, Germany) at a dosage of 10 mg/kg body weight for sample collection. The samples were collected before and after infection on a weekly basis. Three mL of blood from the femoral artery and 1.5 mL of cerebrospinal fluid (CSF) via lumbar puncture were collected. Saliva samples were obtained by placing swabs under the animals tongue for ten minutes to allow for adequate wetting. Thereafter the swabs were placed in dry cryovial tubes. The urine was obtained via a collection apparatus placed on the bottom of the monkey cage and stored in 50 mL falcon tubes. This was done in the early morning prior to sedation. All samples were collected and stored at −20°C.

### 2.5. DNA Extraction

DNA was extracted from serum, urine, CSF, and saliva samples using genomic DNA isolation kits (Zymo Research, USA) as per manufacturer's instructions.

### 2.6. PCR

Amplification of* T. b. gambiense*-specific glycoprotein (*TgsGP*) gene was done using primer sequences as previously described [18]. The PCR reactions (nested) were performed as described [8] using 1 *μ*L of extracted DNA in a 25 *μ*L reaction mixture. The PCR amplification was performed by incubating the samples for 15 min at 95°C followed by 45 cycles of 1 min at 94°C, 1 min at 63°C, and 1 min at 72°C and a final extension at 72°C for 10 min. Thereafter, amplified products were analyzed by electrophoresis in 2% agarose gels. Gels were stained with ethidium bromide (0.5 *μ*g/mL) (Sigma, USA) and viewed under UV illumination. The negative controls: purified DNA from* T. b. brucei* GUTAT1 and* T. b. rhodesiense* IPR001 and distilled water. The positive control was purified* T. b. gambiense* IL3253 DNA.

### 2.7. LAMP

The TgsGP primers as previously described were used [14]. The reaction mixture of 25 *μ*L consisted of 40 pmol of the inner primers, 5 pmol of the outer primers, 20 pmol of the loop primers (Inqaba biotec, SA), 0.8M betaine (Sigma-Aldrich, St. Louis, MO, USA), 2.8 mM dNTPs mix, 1x Thermopol buffer (20 mM Tris-HCl pH 8.8, 10 mM KCl, 2 mM MgSO_4_, 10 mM (NH_4_)_2_SO_4_, 0.1% Triton X-100) (New England Biolabs, UK), and additional 4 mM MgSO_4_, 8-unit* Bst* DNA polymerase (New England Biolabs, UK), double distilled water, and 2 *μ*L of the template DNA. The positive and negative controls were similar to those used in the PCR reaction. The reactions were carried out in triplicate for 80 minutes in a Loopamp real-time turbidimeter LA320C (Eiken Chemical Co., Japan). Increase in turbidity indicates DNA amplification. After the reaction 1/20 dilution of SYBR green I dye (Sigma-Aldrich, St. Louis, MO, USA) was added to confirm the amplification.

### 2.8. Data Analysis

The percentage detection of the different tests and sample was determined and significant differences between the tests calculated using the chi-square (*χ* ^2^) test were determined. The differences were considered statistically significant when *p* < 0.05. The agreement between tests was quantified using Cohen's kappa statistic (*k*). Epicalc of EpiInfo 7 was used.

### 2.9. Ethics

All protocols and procedures used in this study were reviewed and approved by the Institute of Primate Research (IPR) Institutional Review Committee which incorporates Animal Care and Use Committee (IACUC) review (IRC/19/10).

## 3. Results

### 3.1. Parasitological Methods

**Figure 1:**
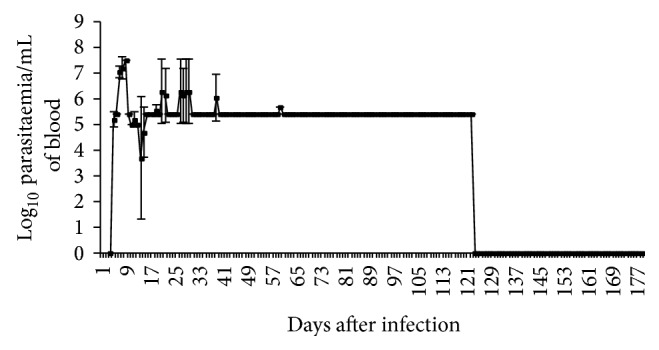
Mean daily parasitaemia of monkeys infected with* T. b. gambiense *IL3253. CSF: there was no CSF parasitosis observed during the experimental period.

The prepatent period was two to three days. The parasitaemia rose to a peak of 10^7^ trypanosomes/mL of blood between 8 and 9 days after infection (dpi). Thereafter, the parasitaemia declined and was characterized by fluctuations to a minimum 2.5 × 10^5^ trypanosomes/mL of blood by 123 dpi. However from 123 to 180 dpi the parasitaemia dropped to undetectable levels (Figure 1).

### 3.2. PCR

**Figure 2:**
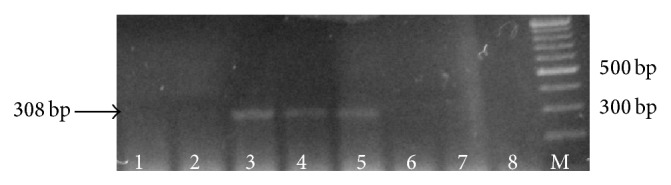
PCR results on gels after electrophoresis. Lane 1 (*Tbb*); Lane 2 (*Tbr*); Lane 3 (positive control* Tbg*); Lane 4 (saliva sample obtained on 14 dpi); Lane 5 (saliva sample obtained 28 dpi); Lane 6 (saliva sample obtained 56 dpi), Lane 7 (saliva sample obtained 70 dpi), Lane 8 (saliva sample obtained 84 dpi), and M (100 bp molecular marker).

The positive control (*T. b. gambiense*) gave the expected 308-base-pair (bp) band. The negative controls (*T. b. brucei* and* T. b. rhodesiense*) were negative (Figure 2).

### 3.3. LAMP

**Figure 3:**
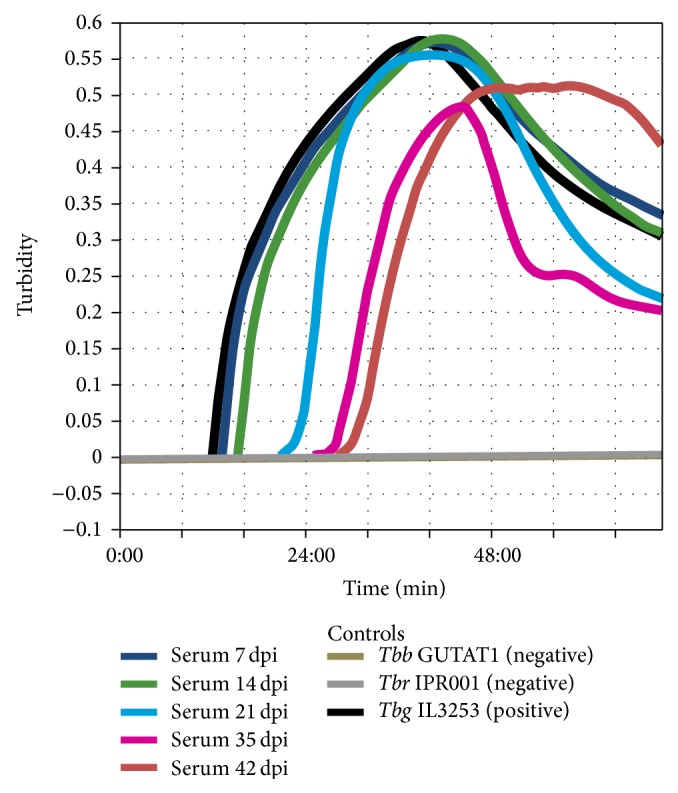
Amplification curves after LAMP reaction in turbidimeter.

**Figure 4:**
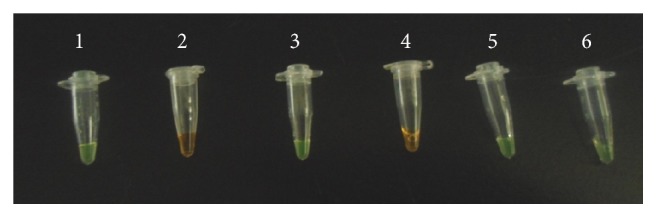
Visual appearance of LAMP results after addition of SYBR green I dye. Green color represents positive reaction while orange represents negative reaction. Tube 1:* Tbg positive* control, Tube 2: negative control (*Tbr*), Tube 3: serum sample obtained 28 dpi, Tube 4: CSF obtained 28 dpi, Tube 5: saliva sample obtained 28 dpi, and Tube 6: urine sample obtained 28 dpi.

Increase in turbidity was noted for the positive control and some samples within 48 minutes of incubation. There was no increase in turbidity in the negative controls as expected (Figure 3). After addition of SYBR green I dye the positive LAMP reactions turned green while the negative ones remained orange (Figure 4).

### 3.4. Comparison between LAMP, PCR, and Parasitological Methods

Parasitological methods detected parasites in the infected monkeys by day 3 after infection (Figure 1). The detection rate gradually dropped and by 119 dpi only 50% detection was obtained. Thereafter the methods could not detect the parasites. Both PCR and LAMP recorded 100% detection in serum samples starting from 7 dpi. LAMP detected trypanosome DNA until 180 dpi but maintained 100% detection up to 133 dpi. On the other hand, PCR could only sustain the 100% detection rate up to 84 dpi. Thereafter the detection dropped and from 161–180 dpi PCR did not detect trypanosome DNA (Table 1).

**Figure 5:**
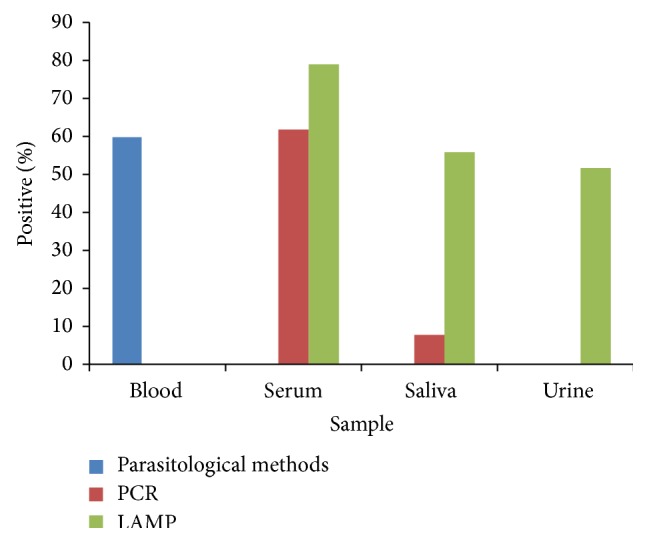
Comparison of LAMP, PCR, and parasitological methods in trypanosome mean detection rate in serum, saliva, and urine samples obtained from monkeys infected with* T. b. gambiense* IL3253 from 3 to 180 days after infection.* There was no amplification in any CSF sample with either LAMP or PCR. There was also no amplification noted in urine using PCR.*

In saliva samples, PCR detected trypanosome DNA from 7 to 63 dpi, thereafter no trypanosome DNA was detected. Between 21 and 77 dpi LAMP recorded 100% detection in the saliva samples. The detection dropped thereafter and from 140 to 180 dpi there was no trypanosome DNA detection. LAMP detected trypanosome DNA in urine samples between days 14 and 126 after infection (Table 1). PCR did not detect trypanosome DNA in the urine samples. Neither PCR nor LAMP detected trypanosome DNA in the CSF samples. Both PCR and LAMP detected parasites beyond the period when conventional parasitological methods did. Trypanosome DNA was detected longest in serum samples followed by saliva and urine (Table 2). The detection rate of LAMP was higher than that of PCR in the serum, saliva, and urine samples (Figure 5).

There was a significant difference in detection between LAMP and PCR (*p* < 0.05) in all the samples. Similarly, the difference in detection between LAMP and parasitological methods was significant (*p* < 0.05). There was good agreement between PCR and parasitological methods in detection of trypanosomes. The difference in detection between both tests was not significant (*p* > 0.05) (Table 3).

## 4. Discussion

The presence of trypanosome DNA in saliva and urine samples is of great significance given the need for noninvasive samples for diagnosis of HAT. The higher sensitivity of LAMP compared to parasitological methods and PCR is also of significance in regard to the search for new diagnostic tests for sleeping sickness. In the current study the performance of LAMP and PCR was assessed in an early stage HAT model [15].

The high number of trypanosomes positive serum samples suggests that the trypanosomes were circulating in the hemolymphatic system. The lack of detection of trypanosomeDNA in the CSF samples may mean that the trypanosomes did not cross the blood-brain barrier and hence late stage disease did not occur. Infected monkeys without trypanosomes in CSF and having WBC counts of less than 5 cells/mm^3^ are regarded as being in early stage of the disease.

PCR performed better than the parasitological methods in monitoring the presence of the infection but, however, it cannot be used as a gold standard in diagnosis of trypanosomiasis because of its challenges to implement in clinical settings. The test was able to detect trypanosome infection for a longer duration compared to the parasitological methods which are regarded as the gold standard. PCR detected trypanosome DNA in the saliva samples during early infection corresponding to the period of high parasitaemia. The lack of detection in urine samples could be due to the presence of inhibitors such as urea and uric acid [19] and elevated acidic conditions that may inactivate the highly sensitive* Taq* DNA polymerase enzyme used in PCR reactions. This enzyme used is also easily inactivated by tissue and blood derived inhibitors [20–22]. Similarly, in a previous study on* T. b. rhodesiense*, trypanosome DNA was not detected in urine samples of infected vervet monkeys [unpublished data]. In this study, all the CSF samples were negative for trypanosome DNA although previous studies have shown that PCR on CSF samples has high sensitivity in staging of HAT [23]. Optimization of reaction conditions is a major setback in development of PCR as a diagnostic tool for HAT. This has especially been noted in samples from serologically positive but aparasitemic patients [9]. In addition, PCR is cost restrictive due to the need for specialized equipment such as automated thermal cyclers and the presence of cold chain to preserve reagents. Thus, recent studies have focused on development of tests such as LAMP which can overcome some of these challenges [10, 24].

LAMP performed better than PCR and parasitological methods as demonstrated by its higher detection rate. In contrast to PCR, LAMP detected trypanosome DNA in urine samples in this study. Indeed, the test detected trypanosome DNA during periods of low parasitaemia when both PCR and parasitology were negative. The significant difference in detection proves that the performance of LAMP was markedly better than that of both parasitological methods and PCR. This is possibly due to the use of* Bst* polymerase that unlike* Taq* polymerase is hardly inhibited by impurities [25]. The detection of trypanosomal DNA in urine and saliva samples is of great value because they are noninvasive samples and are an improvement from the current blood and CSF samples [26].

Trypanosomes have been found to be present in many organs and body fluids. There has been demonstrated localization of* T. b. brucei* in kidney glomeruli of infected rats [27] and* T. lewisi* in kidney capillaries of infected rats [28]. Filtration of the parasite or its DNA may explain the presence of trypanosomal DNA in the urine of the infected monkeys used in this study. However, formation of ammonia in exposed urine causes degradation of DNA and may lower the sensitivity of the tests.

Trypanosome DNA was also detected in the saliva samples of the infected monkeys. It is possible that parasites could have seeped into salivary ducts from either the blood or lymphatic systems. Saliva has a higher pH as compared to urine hence reducing the likelihood of DNA deterioration.

In this study, we targeted TgsGP gene a single gene which encodes a protein specific to the* T. b. gambiense* subspecies and hence ideal for specific detection of* T. b. gambiense *[29]. The LAMP detection rate varied and was 100% in some durations depending on the sample used. The sensitivity appeared to be affected by parasitaemia and hence the amount of DNA in the sample. The concentration of the trypanosomal DNA in the sample was however not assessed. The highest mean detection rate (78.9%) over the entire 180 days experimental period was lower than obtained using a repetitive element (repetitive insertion mobile element, RIME) [30]. A repetitive DNA like RIME means that many copies of DNA are available for amplification and hence greater sensitivity is expected. However, it is important to note that in that study 90% sensitivity was obtained with the samples from parasitological confirmed patients. We recommend that the sensitivity of LAMP targeting the TgsGP gene be further evaluated in clinical samples and especially the noninvasive samples such as urine and saliva. There is also promising development in serological tests. A number of rapid tests are under clinical evaluation in many countries [31]. Combined use of the rapid serological tests and molecular techniques such as LAMP will enhance early diagnosis of HAT in the rural Africa where the disease is endemic.

## Figures and Tables

**Table 1:** Detection (%) of parasitological methods, PCR, and LAMP in serum, saliva, and urine determined at weekly time points in vervet monkeys infected with *T*. *b*. *gambiense*.

DPI	Parasito.	PCR		LAMP		
		Serum	Saliva	Serum	Saliva	Urine
7	100	100	17	100	33	0
14	83	100	83	100	83	17
21	100	100	100	100	100	33
28	83	100	100	100	100	83
35	67	100	100	100	100	100
42	83	100	83	100	100	100
49	83	100	33	100	100	100
56	83	100	17	100	100	100
63	83	100	17	100	100	83
70	67	100	0	100	100	100
77	83	100	0	100	100	83
84	67	100	0	100	83	83
91	50	83	0	100	83	83
98	67	83	0	100	83	67
105	67	83	0	100	83	67
112	50	83	0	100	83	33
119	50	67	0	100	33	17
126	0	83	0	100	33	17
133	0	67	0	100	17	0
140	0	33	0	83	0	0
147	0	33	0	83	0	0
154	0	17	0	83	0	0
161	0	0	0	83	0	0
168	0	0	0	67	0	0
175	0	0	0	67	0	0
180	0	0	0	33	0	0

Key: Parasito. = parasitological techniques; DPI = days after infection.

^∗^There was no amplification in any CSF sample with either LAMP or PCR. There was also no amplification noted in urine using PCR.

**Table 2:** Duration of detection of parasites in blood, serum, saliva, urine, and CSF samples using parasitological methods, PCR, and LAMP in vervet monkeys infected with *T*. *b*. *gambiense* for 180 days after infection (dpi).

Sample	Parasitology	PCR	LAMP
Blood	3–123 dpi (18%)	—	—
Serum	—	7–154 dpi (4%)	7–180 dpi (3%)
Saliva	—	7–63 dpi (11%)	7–133 dpi (5%)
Urine	—	—	14–126 dpi (8%)
CSF	—	—	—

^∗^(1) The percentage of negative samples is given in brackets.

**Table 3:** Comparative analysis of LAMP, PCR, and parasitological techniques in trypanosome detection in serum, saliva, and urine samples obtained from monkeys infected with *T*. *b*. *gambiense* IL3253.

Test	Kappa value	Level of agreement	*χ* ^2^ statistic	*p* value
PCR and parasitology	0.83 (0.36–0.59)	Very good	2.22	0.136
LAMP and parasitology	0.48 (0.36–0.59)	Marginal	26.47	<0.001
LAMP and PCR (serum)	0.59 (0.47–0.72)	Marginal	10.42	0.0012
LAMP and PCR (saliva)	0.14 (0.06–0.21)	Poor	79.79	<0.0001
LAMP and PCR (urine)	0	Poor	6.12	0.0134
